# Ginsenoside Rg1 Prevents Cognitive Impairment and Hippocampus Senescence in a Rat Model of D-Galactose-Induced Aging

**DOI:** 10.1371/journal.pone.0101291

**Published:** 2014-06-30

**Authors:** Jiahong Zhu, Xinyi Mu, Jin Zeng, Chunyan Xu, Jun Liu, Mengsi Zhang, Chengpeng Li, Jie Chen, Tinyu Li, Yaping Wang

**Affiliations:** 1 Department of Histology and Embryology, Laboratory of Stem Cells and Tissue Engineering, Chongqing Medical University, Chongqing, China; 2 The Land Force Lintong Sanatorium, Xi’an, Shanxi, China; 3 Chongqing Stem Cell Therapy Engineering Technical Center, Chongqing, China; Technion - Israel Institute of Technology, Israel

## Abstract

Neurogenesis continues throughout the lifetime in the hippocampus, while the rate declines with brain aging. It has been hypothesized that reduced neurogenesis may contribute to age-related cognitive impairment. Ginsenoside Rg1 is an active ingredient of *Panax ginseng* in traditional Chinese medicine, which exerts anti-oxidative and anti-aging effects. This study explores the neuroprotective effect of ginsenoside Rg1 on the hippocampus of the D-gal (D-galactose) induced aging rat model. Sub-acute aging was induced in male SD rats by subcutaneous injection of D-gal (120 mg/kg·d) for 42 days, and the rats were treated with ginsenoside Rg1 (20 mg/kg·d, intraperitoneally) or normal saline for 28 days after 14 days of D-gal injection. In another group, normal male SD rats were treated with ginsenoside Rg1 alone (20 mg/kg·d, intraperitoneally) for 28 days. It showed that administration of ginsenoside Rg1 significantly attenuated all the D-gal-induced changes in the hippocampus, including cognitive capacity, senescence-related markers and hippocampal neurogenesis, compared with the D-gal-treated rats. Further investigation showed that ginsenoside Rg1 protected NSCs/NPCs (neural stem cells/progenitor cells) shown by increased level of SOX-2 expression; reduced astrocytes activation shown by decrease level of *Aeg-1* expression; increased the hippocampal cell proliferation; enhanced the activity of the antioxidant enzymes GSH-Px (glutathione peroxidase) and SOD (Superoxide Dismutase); decreased the levels of IL-1β, IL-6 and TNF-α, which are the proinflammatory cytokines; increased the telomere lengths and telomerase activity; and down-regulated the mRNA expression of cellular senescence associated genes *p53*, *p21^Cip1/Waf1^* and *p19^Arf^* in the hippocampus of aged rats. Our data provides evidence that ginsenoside Rg1 can improve cognitive ability, protect NSCs/NPCs and promote neurogenesis by enhancing the antioxidant and anti-inflammatory capacity in the hippocampus.

## Introduction

With the growing population and extended lifespan, brain aging becomes a worldwide problem due to its substantial associated disability. For example, one of the strongest risk factors for the Alzheimer’s disease is brain aging. The brain is particularly vulnerable to oxidative stress because of its high oxygen metabolic rate and its relative deficiency in both free-radical scavenging enzymes and antioxidant molecules compared with other organs [1], [2]. During aging, the accumulation of free radicals progressively damages the brain structure and function. Hippocampus is closely related to learning and memory abilities, and as an area where NSCs/NPCs (neural stem cells/progenitor cells) exist in the adult brain, it is of a particular interest in the age-associated neurodegeneration.

*Panax ginseng* has been used as a tonic drug in traditional Chinese medicine for over 2000 years. Ginsenoside Rg1 is one of the most active ingredients of *Panax ginseng*, and has been proven to have various pharmacological actions in anti-oxidation, anti-aging and particularly in memory deterioration [3], [4]. Our previous work has showed a protective anti-aging function of Ginsenoside Rg1 in the neuron system that delays senescence of NSCs/NPCs *in vitro* [5].

To elucidate the function and the underlying mechanism of Ginsenoside Rg1 in age-associated neurodegeneration, we employed the D-gal (D-galactose) induced aging rat model. Chronic systemic exposure of rodents to D-gal induces accelerated aging including deterioration of cognitive and motor skills that are similar to symptoms in natural aging. Therefore, it is regarded and widely used as an ideal model to study the mechanisms and screen drugs for brain aging [6]–[9]. We investigated the effect of Rg1 on spatial memory and hippocampal histopathological damages in the D-gal induced aging rat model. Senescence-associated biomarker, neurogenesis, oxidative stress biomarkers, neuroinflammation biomarkers, telomere shortenting and senescence-associated genes expression in the hippocampus were examined. We propose that ginsenoside Rg1 is able to improve cognitive ability, protect NSCs/NPCs and promote neurogenesis by its anti-oxidative and anti-inflammation capacity.

## Materials and Methods

### Ethics Statement

All experiments were performed in accordance with institutional and national guidelines and regulations and were approved by the Chongqing Medical University Animal Care and Use Committee.

### Animal treatment

Three months old male Sprague-Dawley rats were purchased from the Medical and Laboratory Animal Center of Chongqing and housed in a temperature and light-controlled room with free access to water and food. All surgeries were performed under sodium pentobarbital anesthesia, and all the efforts were made to minimize suffering.

Sixty animals were randomly divided into 4 groups (control, D-gal-administration, Rg1 treatment, and D-gal-administration plus Rg1 treatment). In the D-gal-administration group, D-gal (120 mg/kg·d) was injected subcutaneously daily into rats for 42 days. In the D-gal-administration plus Rg1 treatment group, ginsenoside Rg1 (20 mg/kg·d) was injected peritoneally daily concomitantly for 28 days from day 15 of D-gal injection. All control animals were given saline in the same volume subcutaneously and peritoneally, respectively. In the Rg1 treatment group, saline at the same volumn with D-gal injection was injected subcutaneously for 42 days, and Rg1 (20 mg/kg·d) was injected peritoneally for 28 days from day 15 of saline injection. The body weights were measured every 3 days,there were no significant differences between the groups. The body weight data are provided in the supporting information (Figure S1 in File S1).

### Reagents

Ginsenoside Rg1 (RSZD-121106, Purity = 98.3%) was purchased from Xi’an Haoxuan Biological Technology Co., Ltd (Xi’an, China), dissolved in ddH_2_O at the concentration of 20 mg/ml, and sterilized by ultrafiltration. GSH/GSH-px kit, SOD kit and MDA kit were purchased from Nanjing Jiancheng Bioengineering Institute (Nanjing, China). IL-1β kit, IL-6 kit and goat anti-rabbit secondary antibody were purchased from Wuhan Boster Bio-engineering Co., Ltd. (Wuhan, China). TNF-α Kit was obtained from Uscn Life Science Inc. (Wuhan, China). BCA kit and SA-β-gal Staining kit were purchased from Beyotime Institute of Biotechnology (Shanghai, China). Anti-β-tubulin III antibody was obtained from Sigma Co. LLC. Anti-GFAP antibody was purchased from Wuhan Sanying Biotechnology Inc. (Wuhan, China).

### Morris Water Maze Performances

After the 42-day treatment, spatial memory of the rats was assayed by Morris water maze task. The maze was a tank (80 cm in radius and 45 cm high) filled with water at approximately 24°C. The tank was divided into 4 quadrants, one of which contained a circular escape platform (8 cm in diameter) placed at a fixed position, 2.5 cm below the surface of the water. There were visual cues around the water maze. Oriented navigation trials were performed 4 times per day, for 7 consecutive days with a constant interval of 1 h. In each trial, the animals were gently placed in water in one of the four quadrants, and the starting quadrant was varied randomly over the trials. Rats were allowed a maximum of 90 sec to find the escape platform, where it remained for 30 sec. For all training trials, the time that it took the rat to reach the submerged platform (escape latency) was recorded to assess spatial learning ability. On the eighth day, another set of tests consisting of a 120 sec trial with the platform removed was conducted. Besides escape latency before reaching the platform, time spent in the target quadrant and the number of target crossings over the previous location of the target platform were recorded to assess spatial memory ability. The target quadrant was defined as the quadrant that previously contained the platform, the radius of which was limited to 70 cm in this assessment.

### Detection of oxidation-associated biomarkers

After the therapy, hippocampuses were collected and lysed in ice bath for 30 min. The supernatant was collected after centrifugation (12000 rpm, 4°C, 30 min). GSH-px activity and GSH content, SOD activity and MDA content were detected by chemical colorimetric analysis according to the manufacturer’s instructions.

To detect GSH-Px activity, the enzymatic reaction in the tube, which contained NADPH, reduced GSH, sodium azide and glutathione reductase, was initiated by addition of H_2_O_2_. And the change in absorbance at 340 nm was monitored. Activity is expressed as U/mg protein. To detect reduced GSH content in each group, 1% trichloroacetic acid was added to the lysates. After centrifugation at 10 000×g for 15 min, protein free lysates were obtained. The reaction mixture for determination of GSH content consisted of lysates and 5, 5′-dithiobis-(2-nitrobenzoic acid) (DTNB) 6 mmol/L. The absorbance at 405 nm was monitored for 6 min using a microtiter plate reader (Bio-Rad Ltd, Japan). The content of GSH was calculated as l mg GSH/g protein from the change in the rate of absorbance on the basis of a standard curve.

The assay for total superoxide dismutases (SOD) is based on the ability to inhibit the oxidation of oxymine by the xanthine-xanthine oxidase system. One unit (U) of SOD activity was defined as the amount that reduced the absorbance at 550 nm by 50%, and data were expressed as units per microgram of hippocaumpus protein. Thiobarbituric acid reaction (TBAR) method was used to determine the MDA which can be measured at the wave length of 532 nm by reacting with thiobarbituric acid (TBA) to form a stable chromophore production. MDA content was expressed as nmol per milligram of hippocampal protein. Protein concentration was measured using the method of Bradford. Bovine serum albumin was used as a standard.

### Detection of proinflammatory cytokines in the hippocampus by ELISA

The supernatant was collected as above, and the levels of proinflammatory cytokines IL-1β, IL-6 and TNF-α in the hippocampus in each group were measured by ELISA kit following the manufacture’s instructions. The level of both cytokines was determined by the competitive binding of the cytokines in the samples with ^125^I-radiolabled IL-1β and TNF-a standards respectively. Data were shown as the actual content of the lysates per mg tissue lysates.

### Tissue processing and immunofluorescence

Animals were reanaesthetized and transcardially perfused with 4% buffered paraformaldehyde solution (pH 7.4), then the brains were separated and post-fixed for 4 hours, followed by dehydration in 20% sucrose at 4°C. Free floating sections of 20 µm were cut with a cryotome.

For immunofluorescence analysis, appropriate sections were recovered and washed with TBS with 0.3% Triton X-100 followed by blocking with 1.5% normal goat for 4 h at room temperature. Sections were then incubated with anti-β-tubulin III (a neuron marker), anti-GFAP (an astrocyte marker) and Gal-c (a mature oligodendrocyte marker) antibodies all diluted at 1∶200 for 24 h at 4°C. Later, after TBS washes, sections were incubated with secondary antibodies for 1 h at 37°C. DAPI was used for nuclear staining. Finally, 20 µl of glycerole was applied to each slide and a cover slip was sealed in place. All slides were viewed directly under a fluorescence microscope (Eclipse, Nikon). Controls included omitting the primary and secondary antibodies. The total numbers of cells were estimated on three randomly selected sections taken through the central extent of the dentate gyrus area.

### Senescence-associated *β*-galactosidase cytochemical staining

The SA-*β*-gal (senescence-associated *β*-galactosidase) staining was carried out according to the manufacturer’s instructions. Frozen section preparation procedure described above was used for cytochemical staining. In brief, slides were washed twice by PBS, fixed by Fixative Solution for 15 min at room temperature, and stained by X-Gal Staining Solution (100 mM sodium phosphate, 2 mM MgCl2, 150 mM sodium chloride, 0.01% sodium deoxycholate, 0.02% NP-40, 5 mM potassium ferricyanide, 5 mM potassium ferrocyanide, and 1 mg/ml X-gal, pH 6) for 24 hours at 37°C in dark without CO_2_. After the incubation, sections were washed in PBS and viewed under bright field at 400×magnification. Quantitative image analysis was performed by a blinded observer counting at least 3 random fields. The intensity of SA-*β*-gal-positive cells was evaluated by means of a ROD (relative optical density) value. ROD of SA-*β*-gal-positive cells in CA3 area of the hippocampus was obtained after transformation of mean gray values into ROD using the formula: ROD = log (256/mean gray). Images were collected from at least three different sections per structure and per animal by Image Pro Plus software.

### Cell proliferation analyses

On the day 41 of treatment, BrdU (50 mg kg^−1^ in saline) was administered intraperitoneally three times with an interval of 4 hours. 12 hours after the final administration, the rats (5–6 animals per group) were reanaesthetized and perfused transcardially with 4% PFA in PBS. Hippocampuses were collected and postfixed. Frozen sections and immunofluorescence were performed as previously described. The primary antibody was the mouse monoclonal anti-BrdU (1∶200; Sigma Co. LLC.). The total number of BrdU^+^ cells was estimated on three randomly selected sections taken through the central extent of the dentate gyrus area with a fluorescence microscope (Eclipse, Nikon).

### Western blotting analysis

Hippocampuses in each group were collected after the therapy. Total protein was extracted, and the concentrations were measured by a BCA procedure. Samples containing 50 µg protein were separated on SDS-PAGE and transferred to PVDF membranes. Membranes were incubated overnight at 4°C with the anti-SOX2 antibody diluted at 1 : 500. The secondary antibody was diluted at 1 : 5,000 in TBST. The membranes were visualized using the enhanced chemiluminescence detection system (Pierce, USA). The level of β-actin was used as an internal control. Relative intensities were quantified using Quantity One (Bio Rad).

### RNA extraction and realtime quantitative RT-PCR

Hippocampuses were collected after the therapy. Total mRNA was extracted using TRIZOL Reagent (TaKaRA, Japan), according to the manufacturer’s protocol. OD260/OD280 of RNA: 1.8∼2.0. First-strand cDNA was created by RT (TaKaRA, Japan). Real-time PCR was carried out using BIO-RAD sequence detection system (cfx96). DNA was amplified by an initial incubation at 94°C for 5 min followed by 40 cycles of 94°C denaturation for 15 sec, annealing at 60°C for 60 sec, 72°C extension for 1 min. mRNA expression levels were normalized against *Gapdh* mRNA level and analyzed by the comparative cycle threshold method. The PCR primers used are provided in the supporting information (Table S1 in File S1).

### Measurement of telomere length by Southern blot

Telomere lengths were measured from the hippocampus according to the previously described method [10]. In brief, after extraction, DNA was inspected for integrity, digested, resolved by gel electrophoresis, transferred to a membrane, hybridized with labeled probes and exposed to X-ray film. The telomere lengths were measured by Western Biotechnology Corporation (Chongqing, China).

### Detection activity of telomerase by silver staining TRAP-PCR

The supernatant was collected as above. The concentrations were measured by Coomassie brilliant blue. The PCR reaction mixture contained 5 µl 10× TRAP buffer, 1 µL dNTPs, 1 µl Taq polymerase,1 µl TS primer and 2 µl extract of telomerase, was incubated for 30 min at 23°C for telomerase-mediated extension of the TS primer. The reaction mixture was subjected to 35 cycles at 94°C for 30 sec, 50°C for 30 sec, and 72°C for 90 sec. TRAP reaction products were separated by 10% polyacrylamide gel electrophoresis and detected by SYBR green (Gene, Inc.) staining.

### Statistical analysis

SPSS version 17.0 software was used for statistical analyses. One-way ANOVA was used for comparison of mean values across the groups and multiple comparisons were made by LSD test. Differences were considered significant at *P*<0.05.

## Results

### Ginsenoside Rg1 restored cognitive impairment caused by D-gal administration

**Figure 1:**
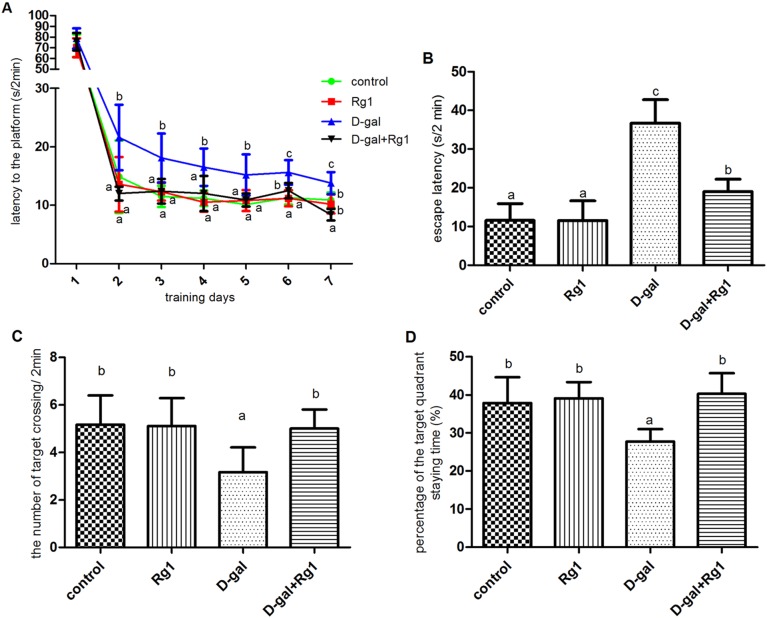
The effect of ginsenoside Rg1 on cognitive impairment caused by D-gal administration (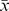
±s, n = 5). The Morris water maze test was carried out to test the spatial learning and memory ability of rats. A. Latencies to find a hidden platform in the water maze during the seven days of place navigation training. On the eighth day, another set of tests was performed when the target platform was removed: B. The escape latency of the rats when the platform was removed. C. The times of rats’ crossing the target quadrant. D. The percentage of time that the rats stayed in the quadrant where the platform was once placed. Different letters represent significantly different values as assessed by ANOVA and LSD tests with P<0.05.

The hidden-platform version of the Morris water maze, a hippocampus-dependent task, requires an animal to learn and remember the relationships between multiple distal cues and the platform location to escape the water [11]. As shown in Figure 1A, rats in the D-gal administration group had significant impairment in spatial learning ability during the seven-day place navigation training because of the longer escape latency compared to the control rats (*P*<0.05); while ginsenoside Rg1 treatment to D-gal administrated rats significantly shortened the escape latency to the similar levels of the control rats. Rats in the Rg1 treatment group showed similar spatial learning ability to that of the control rats during the navigation training.

To assess the spatial memory more directly, the rats were subjected to another trial in which the target platform was removed on the next day after the navigation training. As shown in Figure 1B, for the D-gal administered rats it took longer time to reach the location of the removed platform, and crossed the location fewer times compared to the control group. However, rats in both Rg1 treatment group and D-gal administration plus Rg1 treatment group showed no remarkable differences in the escape latency and target crossing times compared to the control group. In addition, rats in the control group, Rg1 treated group and D-gal administration plus Rg1 treatment group spent more time in the target quadrant than the D-gal administrated rats.

These results indicated that aging model rats had impairments in spatial learning and memory, while the treatment of ginsenoside Rg1 could restore the age-related cognitive impairment caused by D-gal administration.

### Ginsenoside Rg1 reduced the SA-*β*-Gal stainings in hippocampus of brain-aged rats

Aging is known to be associated with a slow decline in brain functions and be accompanied by progressive memory loss, dementia and cognitive dysfunctions. As the hippocampus is closely related to learning and memory ability, we have speculated that the loss of cognitive capacity of the rats in the D-gal administration group is related to the hippocampus aging.

**Figure 2:**
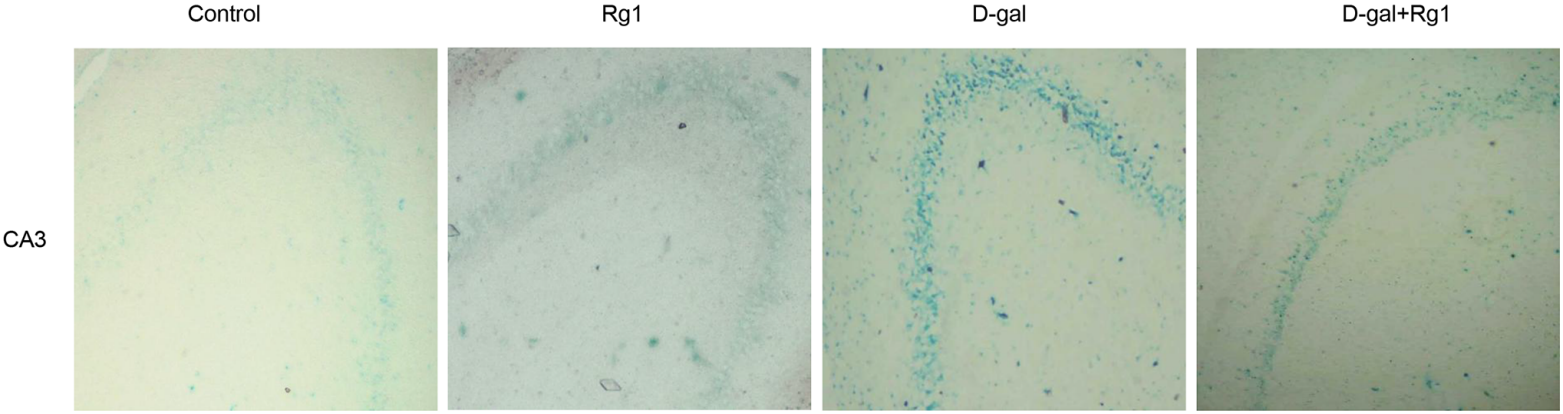
Ginsenoside Rg1 reduced the SA-*β*-Gal stainings in area CA3 in hippocampus of brain-aged rats (×200). The hippocampuses were collected and fixed after the treatment. The SA-*β*-gal (senescence-associated β-galactosidase) staining was carried out on the slides to explore the aging of the hippocampus. The aged cells are stained in blue in the cytoplasm. The intensity of SA-β-gal-positive was evaluated by means of a ROD (relative optical density) value (Table 1).

SA-*β*-gal is one of the most widely used biomarkers for aging cells [12], and aged cells are stained in blue by it in the cytoplasm. As shown in Figure 2, barely any SA-*β*-gal positive cells were observed in DG area among all the groups, while aged cells were observed in the CA3 area. This pattern was consistent with a previous study [13]. The intensity of SA-*β*-gal staining was evaluated by means of a ROD (relative optical density) value. The ROD of the SA-*β*-gal stainingwas not statistically significant between the control group and the Rg1-treated group. Remarkably, D-gal administration induced a remarkable increase in the ROD of the SA-*β*-gal staining, compared to the control group (Figure 2, Table 1). However, in the D-gal administration plus Rg1 treatment group, the ROD was significantly reduced (Figure 2, Table 1). It demonstrates that Rg1 can protect the hippocampus against senescence.

**Table 1:** The effect of ginsenoside Rg1 on the senescence-associated SA-*β*-Gal stainings in area CA3 in the hippocampus of brain-aged rats (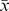 *±s*, *n = 5*).

Group	ROD of SA-*β*-Gal
control	11.8±4.6^a^
Rg1	12.4±6.3^a^
D-gal	67.8±18.67^c^
D-gal + Rg1	36.2±12.46^b^
	P = 0.000

The SA-*β*-gal (senescence-associated β-galactosidase) staining was carried out on the slides. ROD (relative optical density) of SA-*β*-gal-positive cells in CA3 area of the hippocampus was obtained after transformation of mean gray values into ROD using the formula: ROD = log (256/mean gray). Images were collected from at least three randomly selected sections per structure and per animal by Image Pro Plus software. Data are expressed as mean ± SD. Different letters represent significantly different values as assessed by ANOVA and LSD tests with *P*<0.01.

### The effect of ginsenoside Rg1 on the telomere lengths and telomerase activity in hippocampus of brain-aged rats

Telomeres become progressively shortened with each replication of cells and this feature is widely used to evaluate senescence. And the telomerase is responsible for telomere length maintenance. We detected both telomere lengths and telomerase activity in the hippocampus to evaluate the effect of ginsenoside Rg1 on brain senescence.

**Figure 3:**
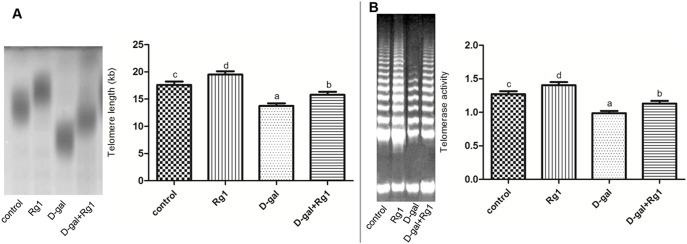
The effect of ginsenoside Rg1 on the telomere lengths and telomerase activity in hippocampus of brain-aged rats (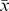
±s, n = 5). A. The effect of ginsenoside Rg1 on the telomere lengths. The DNA of the hippocampus in each group was collected, and telomere lengths were evaluated by Southern Blot. B. The effect of ginsenoside Rg1 on the telomerase activity. The supernatant of the hippocampus in each group was collected and the telomerase activities were detected by silver staining TRAP-PCR. The bar graph indicates quantitative results of telomere lengths and telomerase activity. Different letters represent significantly different values as assessed by ANOVA and LSD tests with P<0.05.

As we expected, both the telomere lengths and the telomerase activity were reduced in the D-gal-administration group, compared with the control, while ginsenoside Rg1 remarkably increased these two parameters in the D-gal administration plus Rg1 treatment group (Figure 3). Moreover, there were no differences between the control group and the Rg1 treatment group (Figure 3).

### Ginsenoside Rg1 promoted neurogenesis in dentate gyrus of hippocampus of brain-aged rats

Although new neurons are generated in the adult hippocampus throughout life by NSCs/NPCs, the rate declines with the increasing age [14]–[17]. Here, we observed the NSCs/NPCs differentiation in hippocampus by immunofluorescence with anti-β-tubulin III (a neuron marker), anti-GFAP (glial fibrillary acid protein, an astrocyte marker) and Gal-c (galactocerebroside, a mature oligodendrocyte marker) antibodies. Though naïve NSCs/NPCs can be marked by GFAP as well, the astrocytes can be identified by the strong expression of GFAP and its morphological character of ramified branches.

**Figure 4:**
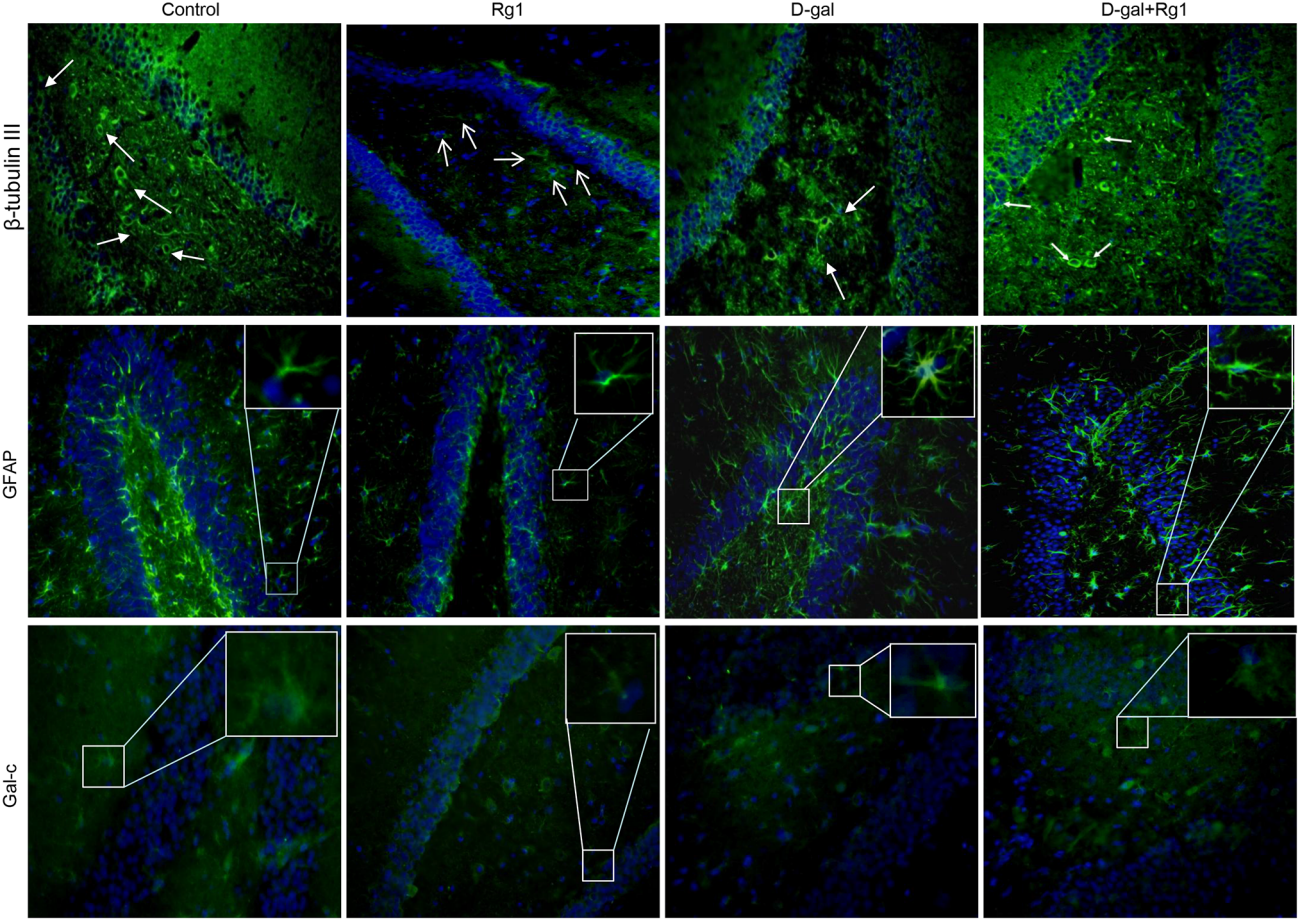
The effect of ginsenoside Rg1 on the NSCs/NPCs differentiation in DG area of hippocampus in brain-aged rats (×400). Hippocampuses in each group were collected and fixed after the treatment. Immunofluorescence was performed on the frozen sections of the hippocampus to visualize neurons, astrocytes and oligodendrocytes. Neurons were positive for β-tubulin III with relatively large and round cell body and less branches. Astrocytes were positive for GFAP with ramified branches. Astrocytes were activated by exhibiting hypertrophy, with very thick, highly ramified and intensely immunostained branches. Oligodendrocytes were positive for Gal-C with a few branches. DAPI was used for nuclear staining (blue). Numbers of the three types of cells in the DG area were analyzed under a fluorescence microscope (Table 2).

Neurons positive for β-tubulin III had a large and irregularly shaped soma and an eccentrically-placed spherical nucleus in the dentate gyrus (DG) area of hippocampus, and oligodendrocytes positive for Gal-c had a few branches, while astrocytes showed activated characteristics by exhibiting hypertrophy, with very thick, highly ramified and intensely immunostained branches in the D-gal-administration group (Figure 4, panel 1). Furthermore, a remarkable decrease of the cells positive for β-tubulin III and a significant increase of the cells positive for Gal-c and GFAP were observed in the D-gal-administration group, compared with the control group and the Rg1 treated group (Figure 4, Table 2). However, in the D-gal-administration plus Rg1 treatment group, the number of cells positive for β-tubulin III increased remarkably, while the number of cells positive for Gal-c and GFAP decreased, compared with the D-gal-administration group (Figure 4, Table 2). This suggests that Rg1 promote NSCs/NPCs differentiation into neurons rather than glial cells in the aged hippocampus.

**Table 2:** The effect of ginsenoside Rg1 on the cell distributions in DG area of hippocampus in brain-aged rats.

Group	β-tubulin III	GFAP	Gal-c	Brdu
control	90.72±20.31^b^	225.42±57.20^a^	13.01±1.73^a^	12.80±3.70^b^
Rg1	98.2±20.0^b^	212.4±36.57^a^	12.54±2.06^a^	14.20±5.54^b^
D-gal	42.12±10.82^a^	351.90±89.30^b^	39.03±5.20^c^	6.80±3.35^a^
D-gal + Rg1	60.48±15.54^a^	265.30±67.33^a^	28.81±3.83^b^	12.40±2.07^b^
	p = 0.000	p = 0.016	p = 0.000	p = 0.039

The immunofluorescence of β-tubulin III (for neuron), GFAP (for astrocyte) Gal-C (for oligodendrocyte) and BrdU (for newly generated cell) was carried out on the slides. The numbers of marked cells were estimated on three randomly selected sections taken through the central extent of the dentate gyrus area. The experiments were performed three times with similar results. Data are expressed as mean ± SD. Different letters represent significantly different values as assessed by ANOVA and LSD tests with *P*<0.05.

### The effect of ginsenoside Rg1 on the expression of SOX2, *Nestin* and *Aeg1* in hippocampus of brain-aged rats

**Figure 5:**
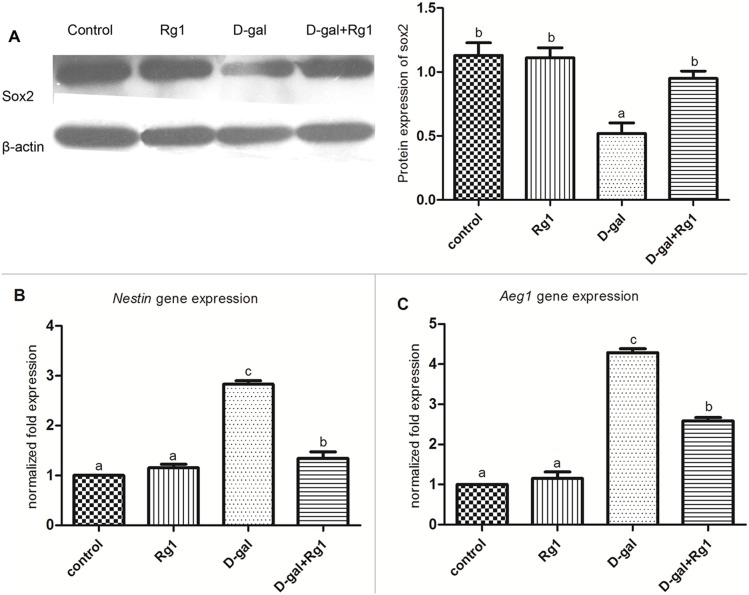
The effect of ginsenoside Rg1 on the expression of SOX2, *Nestin* and *Aeg1* in hippocampus of brain-aged rats. Hippocampuses in each group were collected. A. SOX2 protein expression was detected by western-blot, and β-actin was served as an internal standard. Relative intensities were quantified. B and C. *Nestin* and *Aeg1 mR*NA expression was detected by realtime qRT-PCR. All values were normalized against *Gapdh* and expressed as a percentage of control. The experiments were performed three times with similar results. Data are expressed as mean ± SD. Different letters represent significantly different values as assessed by ANOVA and LSD tests with *P*<0.05.

A continuous decrease in the number of NSCs/NPCs underlies the age-related decline in hippocampal neurogenesis [18]. Commonly used markers for neural stem cells include SOX2 and Nestin. The transcription factor SOX2 is involved in the proliferation and/or maintenance of NSCs/NPCs and in neurogenesis [19]. The intermediate filament protein, Nestin, is expressed predominantly in stem cells of the adult brain and is required for the proper self-renewal of NSCs [20]. We further detected the expression of SOX2 and Nestin to investigate the effect of Rg1 on NSCs/NPCs survival in aged hippocampus. In accordance with our expectation, the protein expression of SOX2 in the D-gal-administration group was significantly lower than that of the control group. Although Rg1 didn’t increase SOX2 expression of the Rg1 treated group relatively to the controls, Rg1 treatment partially rescued the reduction of SOX2 expression in the D-gal-administration plus Rg1 treatment group (Figure 5A). It suggests that the ginsenoside Rg1 can protect NSCs/NPCs in the hippocampus of aged rats.

However, the expression of *Nestin* mRNA was increased violently in the D-gal administration group, and the treatment of Rg1 reversed this D-gal induced increase (Figure 5B). As re-expression of *Nestin* also associated with the astroglial activation during neurodegeneration [21], and the GFAP positive cells in the D-gal administration group showed activated astrocyte appearance (Figure 4), we suppose that the enhanced expression of *Nestin* is due to the activation of astroglials. Therefore, we further explored the expression of *Aeg1* (astrocyte elevated gene-1), a novel modulator of reactive astrogliosis. In accordance with the expression of *Nestin*, the expression of *Aeg1* increased remarkably in the D-gal administration group, and decreased significantly in the D-gal-administration plus Rg1 treatment group (Figure 5C). Additionally, the expression levels of SOX2, *Nestin* and *Aeg1* of the Rg1 treatment group weren’t significantly different from the control group. The results demonstrate that astrocytes are activated by D-gal administration as in early stage of neurodegenetive disease or brain damage; however, astrocytes activation is reduced by Rg1 treatment.

### Ginsenoside Rg1 increases neurogenesis in brain aged rats by increasing new cell number

**Figure 6:**
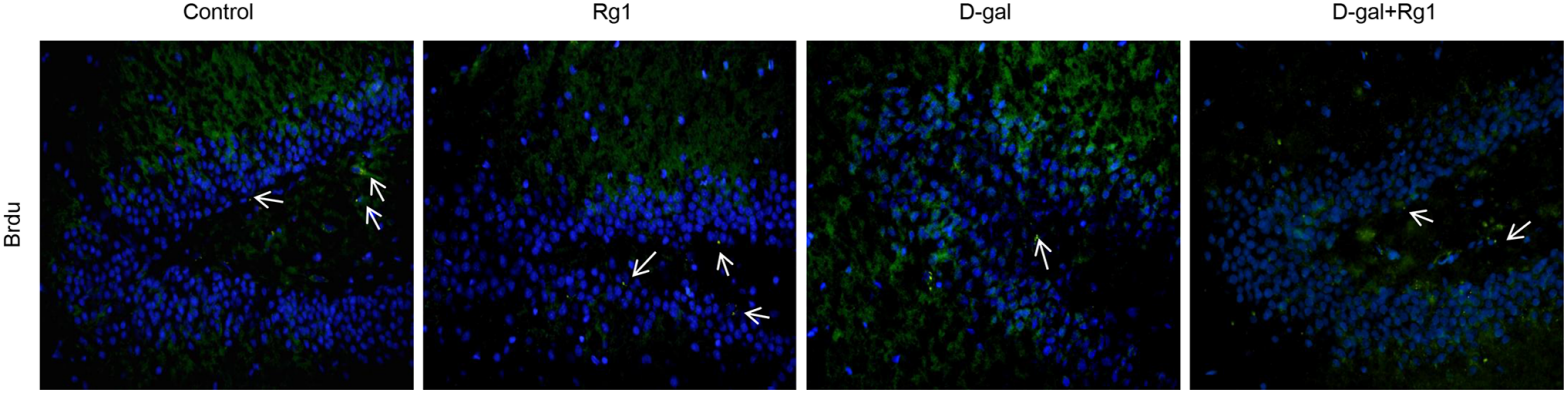
The effect of ginsenoside. Rg1 on cell proliferation in the DG area of the hippocampus of brain-aged rats. BrdU were administrated to the rats in each group three times on day 41 of the treatment to mark the newly generated cells. Hippocampuses were collected on the next day. Frozen slides were incubated with anti-BrdU antibodies. Arrows indicate newly generated cells which are stained by anti-BrdU antibody (green) around the nucleus (blue) during the final 24 hours of the treatment. Numbers of BrdU+ cells in the DG area were analyzed under a fluorescence microscope (Table 2).

To explore whether ginsenoside Rg1 induced new cells production in the hippocampus, the total number of new cells was estimated in the dentate gyrus of all groups using immunofluorescence of BrdU (Figure 6). A student’s t-test confirmed that the total number of BrdU^+^ cells was lower in the dentate gyrus of D-gal administered rats relative to controls (Figure 6; Table 2). Although single treatment with Rg1 could not increase the total new cell number compared with the control, BrdU^+^ cells increased notably in the D-gal-administration plus Rg1 treatment group relatively to the D-gal administered rats (Figure 6; Table 2). It suggests that Rg1 promotes neurogenesis by increasing the number of new cells.

### The anti-oxidative effect of ginsenoside Rg1 on the hippocampus in brain-aged rats

Oxidative stress of ROS (reactive oxygen species) is one of the main causes of cells’ senescence. SOD (Superoxide Dismutase) and GSH-px (glutathione peroxidase) are two important enzymes that participate in the removal of ROS from the cellular environment. MDA (Malondialdehyde) is an end-product of ROS-induced peroxidation, and it is widely used as an oxidative stress biomarker; while GSH is the substrate of GSH-Px and its consumption decreases with age. We evaluated the SOD activity and MDA contents, GSH-Px activity and GSH reduced level in the hippocampus to figure out whether the anti-aging effect of ginsenoside Rg1 was mediated by alleviating oxidative stress.

Compared to the control group, SOD activity, GSH-Px activity and GSH consumption decreased significantly and the MDA content increased remarkably in the hippocampus in D-gal-administration group (Table 3). Meanwhile, Rg1 rescued the reduction of SOD activity, GSH-Px activity and GSH consumed level significantly, and partially rescued the increase of MDA in the D-gal-administration plus Rg1 treatment group (Table 3). Interestingly, Rg1 single treatment also increased the activity of the two anti-oxdative enzymes and the reduced level of GSH, and reduced the MDA content. It shows that ginsenoside Rg1 exerts antioxidant effects against oxidative stress, by enhancing activity of endogenous anti-oxidative defense enzymes.

**Table 3:** The effect of ginsenoside Rg1 on anti-oxidant ability in the hippocampus of brain-aged rats (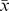
±s, n = 5).

Group	SOD (U/mg prot)	MDA (nmol/mg prot)	GSH (mg/g prot)	GSH-px (U/mg prot)
control	4.52±0.57^c^	1.42±0.17^a^	1.83±0.16^b^	13.58±1.39^b^
Rg1	6.04±0.76^d^	1.12±0.11^d^	2.29±0.20^c^	15.54±1.15^c^
D-gal	2.95±0.37^a^	2.41±0.35^c^	0.55±0.05^a^	10.8±1.62^a^
D-gal + Rg1	3.64±0.46^b^	1.82±027^b^	2.11±0.19^c^	12.5±1.26^b^
	p = 0.000	p = 0.000	p = 0.000	p = 0.000

The supernatant of hippocampus in each group was collected. The anti-oxidative ability was determined by chemical colorimetric analysis of SOD activity and MDA content, and GSH-px activity and reduced GSH level. Data are expressed as mean ± SD. Different letters represent significantly different values as assessed by ANOVA and LSD tests with P<0.05.

### Ginsenoside Rg1 decreased the levels of proinflammatory cytokines of hippocampus in brain-aged rats

Chronic inflammation in the brain is associated with natural aging and neurodegeneration. Increased levels of proinflammatory cytokines as IL-1β, IL-6 and TNF-α have been found in inflammatory tissue and correlated well with aged brain.

The levels of IL-1β, IL-6 and TNF-α increased significantly in the hippocampus of the D-gal-administration group, compared with the control group. However, the levels of the proinflammatory cytokines were reduced notably in the D-gal-administration plus Rg1 treatment group, relatively to the D-gal-administration group (Table 4). Meanwhile, Rg1 single treatment didn’t reduce the levels of IL-1β, IL-6 and TNF-α in the Rg1 treatment group, compared with the controls. It suggests that inflammation in D-gal induced aged hippocampus is alleviated by ginsenoside Rg1 treatment.

**Table 4:** The effect of ginsenoside Rg1 on the levels of of IL-1β, IL-6 and TNF-α in the hippocampus of brain-aged rats (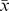
±*s*, *n = 5, pg/mg*).

Group	IL-1β	IL-6	TNF-α
control	0.456±0.019^b^	45.60±8.46^a^	3.885±0.321^a^
Rg1	0.409±0.020^a^	46.43±7.12^a^	3.445±0.286^a^
D-gal	0.620±0.030^c^	61.9±9.90^b^	6.005±0.945^c^
D-gal + Rg1	0.468±0.023^b^	57.96±9.57^b^	5.135±0.426^b^
	p = 0.000	p = 0.008	p = 0.000

The supernatant of hippocampus in each group was collected. The proinflammatory cytokines levels of IL-1β, IL-6 and TNF-α were measured by ELISA kit. Data are expressed as mean ± SD. Different letters represent significantly different values as assessed by ANOVA with P<0.05.

### Ginsenoside Rg1 down-regulated the expression of senescence-associated genes in hippocampus of brain-aged rats

**Figure 7:**
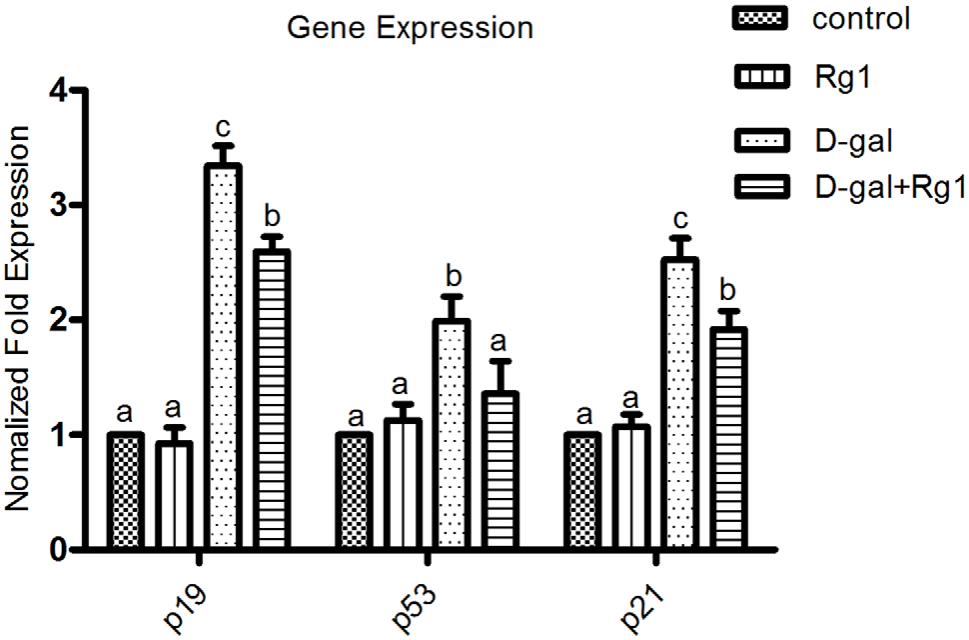
Ginsenoside Rg1 down-regulated the expression of *p19^Arf^, p53, p21^Cip1/Waf1^*mRNA in hippocampus of brain-aged rats. The mRNA of hippocampus in each group was collected. Senescence-associated gene expressions were detected by qRT-PCR. All values were normalized against *Gapdh* and expressed as a percentage of control. The experiments were performed three times with similar results. Different letters represent significantly different values as assessed by ANOVA and LSD tests with *P*<0.05.

*P19^Arf^-Mdm2-p53-p21^Cip1/Waf1^* pathway is a main signal transduction pathway involved in cell aging processes. Therefore, we performed qRT-PCR to explore the mRNA expressions of *p53*, *p19^Arf^* and *p21^Cip1/Waf1^*, which are in the core position of the pathway. The expression of *Gapdh* was used as the inner control. As shown in Figure 7, the mRNA expression of *p53*, *p19^Arf^* and *p21^Cip1/Waf1^* in the D-gal-administration group was violently higher than that of the control group. However, in the D-gal-administration plus Rg1 treatment group, the expression of the genes was significantly lower than that of the D-gal-administration group (Figure 7). Meanwhile, there were no differences between the control group and the Rg1 treatment group. It indicates that ginsenoside Rg1 can down-regulate the expression of senescence-associated genes in the hippocampus of brain-aged rats.

## Discussion

During natural aging, the brain undergoes progressive morphologic and functional changes resulting in the observed behavioral retrogression, such as declines in motor and cognitive performance. It will be of a great value to find out drugs against neurodegeneration to delay brain senescence. It has been reported that an increase of adult hippocampal neurogenesis may have therapeutic potential for reversing impairments in pattern separation and dentate gyrus dysfunction such as those seen during normal aging [22]. In the present study, we demonstrate that ginsenoside Rg1 treatment protects hippocampus against abnormalities in a well-characterized aging rat model by D-gal administration. Rg1 treatment improved hippocampus-associated cognition, promoted NSCs/NPCs differentiation into neurons, and delayed cellular senescence in the hippocampus via anti-oxidant and anti-inflammation ability. D-gal administration model is a mimetic aging model related to free radical and the accumulation of waste substances in metabolism [23]. Similarly, the accumulation of free radicals progressively damages the brain function during natural aging, and D-gal-administrated rodents mimic many characteristics of the normal brain aging process. Therefore, D-gal-induced aging model is regarded as an ideal mimetic aging model to study the mechanism related to the brain aging and screen drugs for brain aging [24]–[26]. Furthermore, enhancing endogenous antioxidants is now widely regarded as an attractive therapy for conditions associated with mitochondrial oxidative stress, and ginsenoiside Rg1 is widely reported as having anti-oxidation effect [3]–[5]. Therefore, we employed D-gal administration model in this study as we established it previously [27]. In this study, we induced aging by D-gal administration and observed significant reduction in spatial memory, cell proliferation, and neurogenesis in the dentate gyrus. This result was supported by previous studies showing that proliferating progenitor cells were significantly decreased in the seventh week after D-gal administration [28], [29]. In addition, D-gal can induce behavioral impairment in C57BL/6J mice [30] and decrease spatial preference for the target quadrant in the Morris water maze test [31].

We administered ginsenoiside Rg1 to control and D-gal mice and probed spatial memory and learning ability using a water maze test. The administration of ginsenoiside Rg1, a main active component of *Panax ginseng*, significantly reduced the escape latency in the D-gal group, while Rg1 administration to control mice did not significantly change the escape latency. In addition, the administration of Rg1 to D-gal-induced aging mice significantly improved the deficits in platform crossings in probe trial and spatial preference for the target quadrant. This result was coincided with a previous study that Rg1 has profound neuroprotective effects in an Alzheimer mouse model [32].

Rg1 administration to D-gal-induced aging mice significantly increased SA-*β*-gal expression and telomerase, and decrease telomere length in the hippocampus compared to that in the D-gal group. SA-*β*-gal, which reflects the function of the lysosomes, accumulates in aging cells as the lysosomes begin to malfunction. A telomere is a region of repetitive nucleotide sequences at the end of a chromatid. Telomere shortening can limit stem cell functions and regeneration during aging [33]. Both of the biomarkers are widely used to evaluate senescence. It indicates that ginsenoside Rg1 is able to protect against the senescence of the hippocampus, which is coincident with our previous study of the effect of ginsenoside Rg1 on NSCs/NPCs senescence *in vitro* [5].

Aging is associated with a continuous decline in the neurogenesis in the DG area of the normal hippocampus, because of the age-driven disappearance of NSCs/NPCs via their conversion into mature hippocampal astrocytes [18]. Therefore, we propose that the anti-aging effects produced by Rg1 also correlate with increased neurogenesis. Our data in this study supported this hypothesis. Four weeks of Rg1 treatment promoted NSCs/NPCs differentiation to neurons rather than glial cells, because the number of the cells positive for β-tubulin III increased and that of the cells positive for GFAP and Gal-C decreased, compared with the D-gal administration group. β-tubulin III is widely regarded as a neuronal marker in developmental neurobiology and stem cell research [34]. Given the potential significance of new neurons for cognitive function, it has been hypothesized that reduced neurogenesis may contribute to age-related cognitive impairment [18]. The promoted neurogenesis of Rg1 treatment in this study supports the effect of ginsenoside Rg1 in improving cognitive ability (Figure 1) and the function of *Panax ginseng* in preventing memory deterioration. GFAP is highly expressed by astrocytes and is widely used as a marker for differentiated astrocytes, while evidence also indicates that GFAP is expressed by developing NSCs/NPCs [35]. However, in the D-gal administration group of this study, GFAP-positive cells showed morphological characteristics of activated astrocytes. Considering that the activated astrocytes have been identified as a major brain-derived source of inflammatory cytokines [36], and elevated levels of IL-1β (Table 4) can lead to astrocytes activation in a positive feedback way [37], we believe that most GFAP positive cells represent astrocytes in this study. In addition, the treatment of Rg1 significantly decreased the GFAP-positvie and Gal-C –positive cells number. Our results suggest Rg1 can counteract the age-driven NSCs/NPCs deletion and excess astrogenesis and promote NSCs/NPCs differentiation into neurons.

We further examined whether Rg1 promoted neurogenesis by maintaining the NSCs/NPCs. We employed the wide-spreading NSCs/NPCs markers SOX2 and Nestin. The increase of SOX2 level in the Rg1 treatment plus D-gal administration group indicated that the Rg1 could protect NSCs/NPCs survival against D-gal induced aging (Figure 5A). The increase of newly generated cells indicated by BrdU in the Rg1 treatment plus D-gal administration group further revealed the NSCs/NPCs protective effect of Rg1 (Figure 6). Moreover, in NSCs/NPCs, telomeres shortened with age and that telomerase-deficient mice exhibited reduced neurogenesis [38]. In this study, the SOX2 expression was well correlated with the changes of lengths of telomeres and the activity of telomerase in each group. These results suggest that ginsenoiside Rg1 effectively protect NSCs/NPCs survival against D-gal induced aging.

Interestingly, *Nestin* expression increased in the D-gal administration group. Nevertheless, this observation was consistent with the bulk of studies suggesting that in pathological conditions adult glial cells are induced to revert to a more primitive glial form, so that earlier stages phenotypic features, including Nestin, were transiently re-expressed [20], [39], [40]. Another study also illustrated that Nestin was re-expressed in the activated astroglial in the damaged brains [21]. Altogether with the elevated levels of *Aeg1* (Figure 5C) which plays a novel role in mediating reactive astrogliosis and responses to pathogenic and aging factors, it indicates the astrocytes activation in the D-gal-induced aged brain. Moreover, for the increased levels of *Aeg1*, it was also consistent with the morphological characteristic of activated astrocytes in the D-gal-administration group (Figure 4). However, *Nestin* and *Aeg1* expression was down-regulated in the D-gal-administration plus Rg1 treatment group, indicating that the reactive astrogliosis induced by D-gal was alleviated by Rg1 treatment. This result further illustrated that Rg1 could protect the age-related NSCs/NPCs survival and reduce astrogenesis. Cognitive capacity was improved, neurogenesis was restored and reactive astrogliosis was attenuated by ginsenoside Rg1 treatment to D-gal administered rats. We did not elucidate the direct mechanism of Rg1 on these effects in the hippocampus. However, we assumed two possible mechanisms for these effects. One of these is the antioxidant function of Rg1 on the hippocampus, because the oxygen metabolism of D-gal produces many reactive oxygen species (ROS) and may impair learning and memory directly or indirectly [31]. In addition, ROS can potently inhibit neurogenesis and particularly NSCs/NPCs proliferation [41]. Oxidative damage can also affect glial cells, which are connected to neuronal death or decreases in neuronal proliferation [42]. In the present study, Rg1 treatment protected the hippocampus against oxidative stress by promoting the activities of SOD and GSH-Px, which are important anti-oxidative enzymes to remove the oxidative stress accumulated in aging. On the other hand, as the telomere is highly sensitive to the oxidative stress, the increased telomere length and telomerase activities in the D-gal-administration plus Rg1 treatment group may also be due to the effect of the anti-oxidant function of Rg1. These results suggest that ginsenoiside Rg1 effectively attenuates D-gal-induced oxidative damage in the hippocampus, possibly by eliminating free radicals through activating antioxidant enzymes. It should be noted that the activities of SOD and GSH-Px were remarkably increased in the Rg1 single-treated rats. It confirmed the anti-oxidative effect of ginsenoside Rg1, which was consistent with previous studies [43], [44].

Oxidative stress has been implicated in proinflammation [33], and aging is also associated with inflammation [45]. When chronic inflammation occurs in the aged brain, a variety of neurotoxic products and proinflammatory cytokines such as IL-1β, IL-6, and TNF-α are released [45]–[48]. In the present study, Rg1 treatment significantly reduced the levels of IL-1β, IL-6, and TNF-α, compared with the D-gal administration group. It suggests that Rg1 can protect the hippocampus from age-induced chronic inflammation. Furthermore, the elevated levels of proinflammatory cytokines could also be a consequence of astrocytes activation. Sustained activation of astrocytes releases high amount of NO (nitric oxide) and proinflammatory cytokines which accumulate in aging process, to exacerbate neuronal impairments [49], [50]. In this study, the attenuated activation status of astrocytes by the Rg1 therapy may be a combined outcome of the anti-inflammation and the neurogenesis-promoting capacity of ginsenoside Rg1.

Another possible mechanism is associated with p19^Arf^/MDM2/p53 signaling pathway because it is an important signaling pathway controlling senescence [51]. The accumulated P53 protein can transcriptionally activate *p21^Waf1/Cip1^* [51], [52] and other putative effectors [53] that inhibit various kinds of cdk-cyclin complexes. On the other hand, p53 has been shown to mediate all adverse effects of telomere attrition on cell cycle arrest and/or apoptosis in proliferative cells, including stem cell populations. These effects of telomere dysfunction on NSCs/NPCs, including those on neuronal differentiation and neurogenesis, are mediated by activation of *p53* [51]. In the present study, the reduced mRNA levels of *p53*, *p19^Arf^*, *p21^Cip1/Waf1^* by Rg1 indicated that Rg1 regulates the expression of the genes to delay telomere shortening and hippocampus senescence.

Adult NSCs/NPCs exist in the hippocampus in mammals, and neurogenesis continues throughout the lifetime. Recent study suggests [54] that promoting neurogenesis in adult mammals might provide a therapeutic way to cure age-associated neurodegenerative diseases or to improve age-related cognitive impairment. In the present study, we provided evidence that ginsenoiside Rg1 treatment can prevent cognitive impairment and hippocampus senescence in a rat model of D-galactose-induced aging, suggesting Rg1 is involved in the anti-oxidation and anti-inflammation regulating telomere length, NSCs/NPCs survival and differentiation. These effects may serve as the elementary mechanism underlying nootropic and anti-aging actions of ginsenoside Rg1.

## Supporting Information

File S1 (DOC)
