# Supplementary material for: Ginsenoside Rg1 Prevents Cognitive Impairment and Hippocampus Senescence in a Rat Model of D-Galactose-Induced Aging
Source: PLoS One. 2014 Jun 30;9(6):e101291. doi: 10.1371/journal.pone.0101291 (PMC4076296; doi:10.1371/journal.pone.0101291)
Supplement: File S1 — (DOC) [file pone.0101291.s001.doc]

**supporting information**


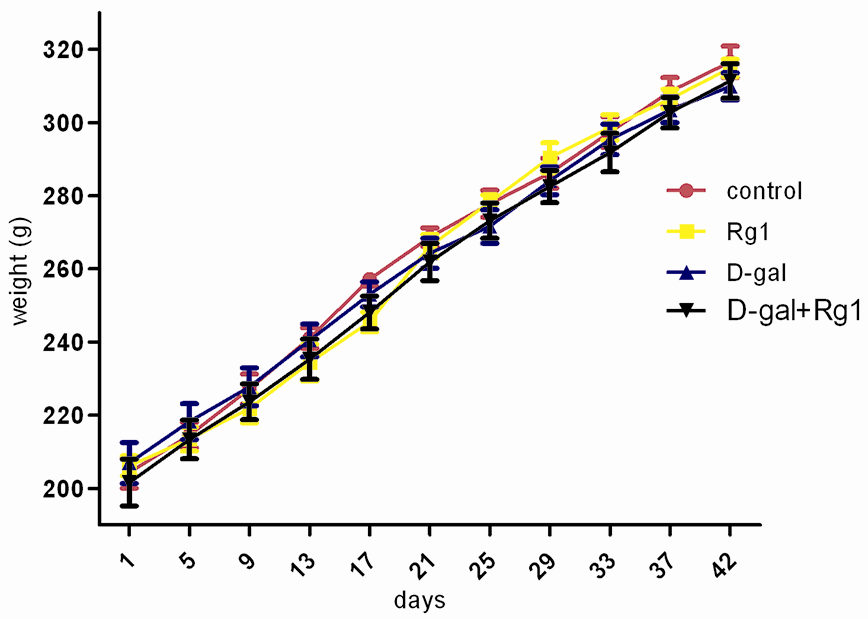


**Figure S1. The body weight of rats during the treatment.** The body weight of each rats was measured every 3 days. The body weight increased gradually during the treatment; however, there were no significant differences among the three groups.

| **Primer** | **Forward（5'-3'）** | **Reverse（5'-3'）** |
| --- | --- | --- |
| P19^ARF^  P53  P21^CIP1/WAF1^  Aeg-1  Nestin  GAPDH | 5’-ACCCCAAGTGAGGGTTTTCT-3’  5’-GCCATCTACAAGAAGTCACAGC-3’  5’-GTGATGTCCGACCTGTTCC-3’  5’-TGCCGAGCCATCTGTTACTT-3’  5’-GGTAGGGCTAGAGGACCCAA-3’  5’-CCCACGGCAAGTTCAACGGCA-3’ | 5’-GATCCTCTCTGGCCTCAACA-3’  5’-GATGATGGTAAGGATAGGTCGG-3’  5’-GCAAAGTTCCACCGTTCTC-3’  5’-TCAGACTTGGTCTGTGAGGGA-3’  5’-TGGGCAATTCAAGGATCCCC-3’  5’-TGGCAGGTTTCTCCAGGCGGC-3’ |

**Table S1. The primers sequences.**
